# The Two-Track Investigation of Fibronectin Binding Protein A of *Staphylococcus aureus* from Bovine Mastitis as a Potential Candidate for Immunodiagnosis: A Pilot Study

**DOI:** 10.3390/ijms24076569

**Published:** 2023-03-31

**Authors:** Anna Dobrut, Agata Młodzińska, Kamil Drożdż, Dagmara Wójcik-Grzybek, Katarzyna Michalak, Dorota Pietras-Ożga, Jolanta Karakulska, Katarzyna Biegun, Monika Brzychczy-Włoch

**Affiliations:** 1 Department of Molecular Medical Microbiology, Chair of Microbiology, Faculty of Medicine, Jagiellonian University—Medical College, 31-008 Kraków, Poland; 2 Bioidea Company, 02-991 Warsaw, Poland; 3 Department of Experimental Physiology, Chair of Physiology, Jagiellonian University—Medical College, 31-008 Kraków, Poland; 4 Department of Epizootiology and Infectious Diseases Clinic, University of Life Science in Lublin, 20-033 Lublin, Poland; 5 Department of Microbiology and Biotechnology, Faculty of Biotechnology and Animal Husbandry, West Pomeranian University of Technology in Szczecin, 70-311 Szczecin, Poland

**Keywords:** fibronectin binding protein A, mastitis, immunoreactive protein, bioinformatic analysis, immunodiagnostics

## Abstract

Bovine mastitis is the most common disease affecting dairy cattle worldwide and it generates substantial losses for cattle breeders. One of the most common pathogens identified in infected milk samples is *Staphylococcus aureus*. Currently, there is no fast test for recognizing bacteria species on the market. The aim of this study was to bioinformatically and laboratory detect and characterize the fibronectin binding protein A (FnBPA) of *S. aureus* (SA) in milk samples obtained from cows diagnosed with mastitis. More than 90,000,000 amino acid sequences were subjected to bioinformatic detection in the search for a potential biomarker for bovine SA. The analysis of FnBPA included the detection of signal peptides and nonclassical proteins, antigenicity, and the prediction of epitopes. To confirm the presence of the *fnb*A gene in four SA isolates, amplification with specific primers was performed. FnBPA was detected by immunoblotting. The immunoreactivity and selectivity were performed with monoclonal anti-FnBPA antibodies and SA-negative serum. The bioinformatic analysis showed that FnBPA is a surface, conservative, immunoreactive, and species-specific protein with antigenic potential. Its presence was confirmed in all of the SA isolates we studied. Immunoblotting proved its immunoreactivity and specificity. Thus, it can be considered a potential biomarker in mastitis immunodiagnostics.

## 1. Introduction

Bovine mastitis constitutes the most common clinical problem in veterinary treatment and generates losses in the millions for the dairy industry [1]. It is estimated that the frequency of the clinical form of mastitis ranges from 12% to 30% [2]. In turn, the economic losses generated by clinical forms of mastitis can even reach EUR 240 per cow per year [3]. These losses stem from reduced volumes of milk, lower milk quality and the need to isolate sick animals—during which time they do not produce milk and require appropriate treatment [1]. The most common pathogens responsible for mastitis are the bacteria *Staphylococcus aureus*, *Streptococcus uberis*, *Streptococcus dysgalactiae*, *Corynebacterium bovis*, *Mycoplasma bovis*, *Escherichia coli*, and *Klebsiella pneumoniae* but fungi and algae cause it as well [4,5].

The gold standard in the diagnosis of mastitis are methods based on somatic cell count (SCC) in quarter samples [6]. The undoubted advantage of this method is the ability to quickly identify an infection directly in the field; however, it does not identify the etiological factor that caused the infection. This knowledge is crucial when introducing targeted antibiotic therapy, which, in opposition to empirical therapy, can slow the adverse phenomenon of increasing resistance to antibiotics [7,8]. Therefore, a notable trend in the investigation of alternative diagnostic methods is to emphasize molecular and serological methods. The best-known method for molecular detection of pathogens in milk and other biological samples is polymerase chain reaction (PCR). It can detect even a small amount of biological material due to DNA amplification, which is the key to this procedure [9]. As an alternative to molecular methods, serological methods based on the antigen–antibody reaction are gaining in popularity. Enzyme-linked immunosorbent assay (ELISA) is one example. This method allows quantitative or semiquantitative detection of antigens or antibodies in samples with significant selectivity and specificity and with a shorter waiting time for the results than classical cultivation methods [10]. However, the need to carry out both molecular and serological methods under laboratory conditions is a key limitation considering the nature of the work of veterinarians. Thus, the most desirable diagnostic product should be possible to perform next to the animal without a laboratory, rapid, easy to perform and interpret, and inexpensive. An example of a test that demonstrates the mentioned characteristics is lateral flow assay (LFA), which is based on the detection of specific bacterial surface proteins (biomarkers) [9,10,11].

In immunodiagnostics, the determination of a suitable biomarker is crucial. It should demonstrate the following features, among others: it should be species specific, so as to exclude false positive results; conservative among species, so as to exclude false negative results; and immunoreactive, in order to be detected by the antibodies. The determination of a suitable biomarker requires a combination of two complementary methods: bioinformatic analysis and in vitro techniques. Bioinformatic analysis allows one to identify potential candidates for biomarkers according to their desired features: bacterial species specificity, cell localization, immunoreactivity, and other functional or structural features. They also narrow down the pool of candidates for further in vitro studies, the purpose of which is to verify under laboratory conditions obtained in silico results. The synergy of these methods shortens the study time while maintaining the reliability of the results [12]. One protein that demonstrates these features is fibronectin binding protein A (FnBPA) [13]. This ~120 kDa adhesin belongs to cell-wall-anchored proteins (CWA) and is one of the virulence factors responsible for the infection caused by *S. aureus*. FnBPA and the closely related FnBPB possess and A domain, which is involved in binding ligands such as fibrinogen, elastin, and plasminogen. Both proteins belong to the group of microbial surface components recognizing adhesive matrix molecule (MSCRAMM) group [14]. FnBPA is also known for its immunogenic role [15,16,17,18,19].

The aim of our paper was to study bioinformatically and laboratory-detected fibronectin binding protein A in *S. aureus* obtained from milk from cows diagnosed with mastitis, which thank to its immunoreactivity, specificity, conservativity and localization in bacterial cells can be considered a potential biomarker in LFA for bovine mastitis diagnostics.

## 2. Results

### 2.1. Bioinformatic Analysis

**Figure 1:**
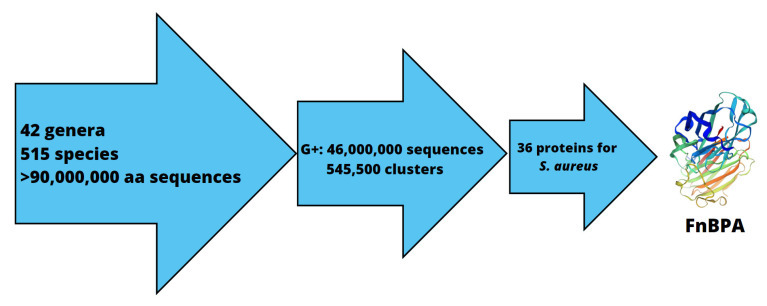
Scheme showing the stages of analysis leading to the identification of FnBPA for *S. aureus*, as a potential biomarker in an immunodiagnostic assay.

In summary, 515 bacterial species, which according to the literature can be detected in cow’s milk and 93,800,000 amino acid sequences collected from the Ensembl Bacteria and UniProt databases were subjected to bioinformatic analysis (Figure 1), among which 292 species and 46,180,426 amino acid sequences were classified as Gram-positive. CD-HIT generated 545,500 clusters corresponding to Gram-positive bacteria at a 70% identity level. Using PSORTb to determine proteins among cell localization, and then combining with the results from CD-HIT yielded 101 proteins representative of *S. aureus*. In this pool, 74 of the proteins were classified as potentially present in bovine milk, as predicted by the NCBI BLAST database. Eventually, 36 surface proteins specific to *S. aureus* at a 75% identity level were identified fibronectin binding protein A (NCBI accession number: WP_001566816.1) was one of them.

FnBPA was also subjected to bioinformatic analysis in order to determine immunogenicity and functionality. In the first stage of the analysis, the protein was classified in terms of its location in the cell and was found in the extracellular space (Busca score: 0.97) with GO:0005615 and in the cell wall (Psortb3.0, score 10). In the next step, FnBPA was used to predict whether it was a classical protein. As a result, a signal peptide (Sec/SPI) was identified, the presence of which determined this protein to be classical (likelihood of SignalP6.0 0.82). Furthermore, the protein in question was classified in terms of antigenicity, leading to its designation as an antigen (VaxiGen score 0.78). The structure of FnBPA was analyzed to identify B cell epitopes, yelding 19 epitopes. The presence of B epitopes allowed further identification of conformational epitopes in the protein. First, the most identical structure of the PDB protein (template) was identified and a protein-template model was built. The template identity was 68.5% with a GMQE of 0.23. The constructed model was then used to identify linear and discontinuous conformational epitopes. As a result of the analysis, 10 linear epitopes and 5 discontinuous epitopes were identified. A low GMQE value of 0.23 found during template investigation may suggest that there is no identical structure of this protein in the PDB database. The closest homologue found was the fragment of chain 1 A of protein rFnBPA (189–505) in a complex with the C-terminal peptide of the fibrinogen gamma chain (PDB ID 4B60.1.A).

### 2.2. Gene Detection

**Figure 2:**
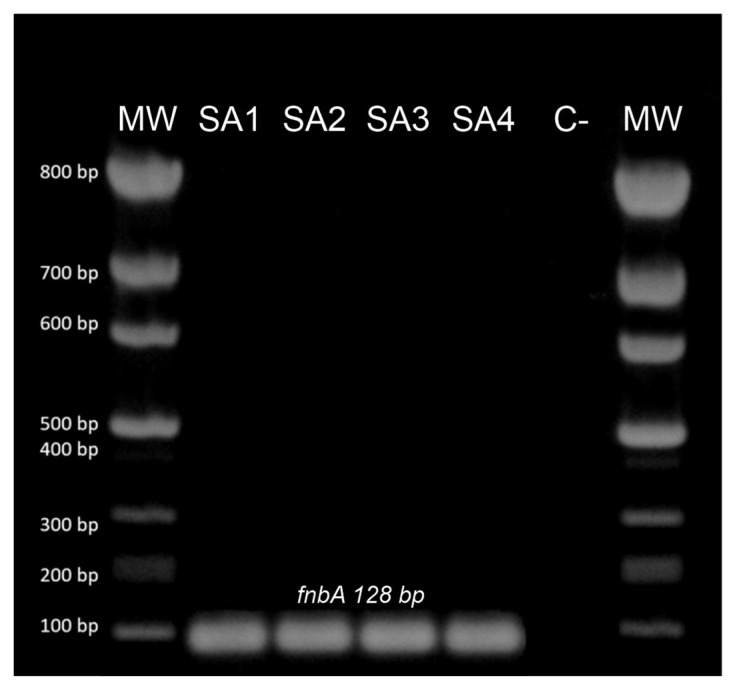
Exemple result of *fnb*A (128 bp) for the detection of the *S. aureus* gene. Legend: M—DNA marker (A&A Biotechnology); SA1, SA13, SA31—examined *S. aureus* isolates from bovine mastitis; C—negative control (DNAse-free water); bp—base pair.

The PCR reaction followed by electrophoresis revealed the presence of the *fnb*A gene in all of the *S. aureus* studied strains (*n* = 4; 100%) (Figure 2).

### 2.3. SDS-PAGE and Immunoblotting

**Figure 3:**
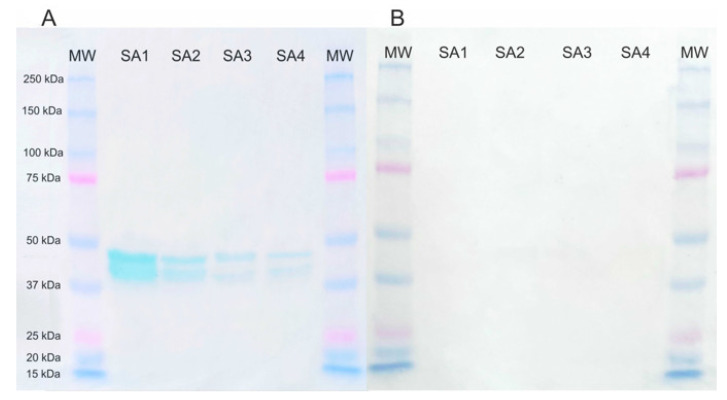
Results of immunodetection of the FnBPA protein fragment in the presence of monoclonal anti-FnBPA antibodies (**A**) and example result of the negative control in the presence of the bovine milk of a healthy cow (**B**). Legend: MW—molecular mass marker (BIO-RAD); SA1–SA4—protein lysates of bovine *S. aureus* isolates.

The electrophoresis of the *S. aureus* protein homogenate allowed us to separate several protein bands, among which only one reacted with anti-FnBPA mAb, confirming the presence of FnBPA among all examined strains, as well as its immunoreactivity. In turn, the lack of immunoreactivity with serum samples obtained from healthy animals confirmed its specificity. The mass of the protein-mAb complex corresponded to approx. 40 kDa molecular mass marker (Figure 3). The band visible in Figure 3 is only a fragment of the whole FnBPA protein, which after probable protein degradation, demonstrated epitopes that specifically recognized anti-FnBPA antibodies.

## 3. Discussion

*S. aureus* is one of the most common agents responsible for the onset of bovine mastitis. This bacterium demonstrates the ability to enter the mammary gland through the teat canal and after adapting to the udder environment it starts to multiply quickly [20]. There are many virulence factors that can be responsible for the pathogenesis of *S. aureus* mastitis. For example, extracellular matrix proteins and their ability to adhere to epithelial cells play an important role in early stages of the infection process by protecting them from flowing out of the gland during the milking process [20,21].

FnBPA is responsible for binding fibronectin (a disordered repeat-region) in the organism of the host [22]. Although fibronectin is not detectable at the luminal site of ductular and secretory epithelial cells in the bovine mammary glands, it is present in the basement membrane in the epithelial cell layer and around myoepithelial cells and fibroblasts [20]. FnBPA as a virulence factor demonstrates immunoreactivity, so it can be considered a good prognostic candidate for use as a biomarker in the immunodiagnostics of bovine mastitis caused by *S. aureus*.

The development of bioinformatics in recent years has had a great impact on proteomic studies. It has enabled the investigation of proteins according to their features and the results can be found in studies focused on immunodiagnostic assays or innovative drugs. In our study, bioinformatic analysis allowed us to isolate one protein among over 90,000,000 amino acid sequences. The protein, fibronectin binding protein A, due to its desirable features, such as specificity, immunoreactivity, and external localization among bacterial cells was included in further investigations with the objective of confirming the in silica results under in vitro conditions. For this purpose, 4 representative *S. aureus* isolates were subjected to immunoblotting to confirm the conservativity and immunoreactivity of FnBPA. It was found that both the *fnb*A gene and the FnBPA protein were present in a pool of isolated proteins from *S. aureus* originating from bovine mastitis. It is also worth noting that the FnBPA protein did not show any reactivity with negative sera obtained from healthy animals.

A surface FnBPA protein is involved in pathogenesis. Characteristic of the C-terminal part of this protein, it comprises a long and intrinsically disordered domain composed of 11/10 fibronectin-binding repeats, which promotes a host’s cell invasion through interaction with the integrin α5β1 via a Fn-mediated bridge. The A domain of FnBPA participates in a homophilic reaction between cells, leading to biofilm formation and enhancing platelet aggregation [13]. FnBPA expression is detectable on the cell surface during the exponential growth phase, which is associated with its function in adhesion and is characteristic of MSCRAMMs. Thus, transcription of the *fnb*A gene is predominant during the exponential phase, while during the stationary phase, FnBPA can be degraded by proteases and shed to the cultivation medium. However, a sufficient amount of this protein remains on the surface of the bacterial cell, thus still allowing it to be detected [23,24,25]. It can be concluded that FnBPA can be considered a good biomarker in detection of *S. aureus* regardless of the growth phase of the bacteria. Moreover, sequence homology between FnBPA and other proteins characteristic of *S. aureus*, fibrinogen binding, and clumping factor A (clfA) was found. The binding site (the C-terminal segment of the γ chain) in Fbg for these proteins is the same, and this affinity indicates a similar mechanism of Fbg binding, which is the DLL (Dock, Lock and Latch) [26,27].

The A domain of FnBPA had been shown to demonstrate high immunogenicity in mice vaccinated with it. Furthermore, the B-cell epitope (IETFNKANNRFSH) has been identified in this domain, and its ability to induce the protective immune response in mice after vaccination with FnBPA as an antigen has been demonstrated. This indicates that the A domain can be considered a promising component of a vaccine to protect against streptococcal infections [13,28]. Interestingly, the C-terminal Fn-binding domain showed weak antigenic properties [29]. In our investigation, strong immunoreactivity was shown. Bands of interest corresponding to the FnBPA protein fragment, were detectable not only in presence of the TMB but also highly reacted in the chemiluminescence method.

Li et al. [15] carried out an investigation on synthetic epitopes (mimotopes) of FnBPA by phage display to induce immunogenicity in mice, single and mixed, and the results reached as high as 66.67% (survival of mice immunized with phage clones displaying a mimotope). The immunogenic role of this protein was also described for *S. aureus* infections in humans [16]. As the economic and epidemiological impact of bovine mastitis on cattle breeders is known, research on protective vaccines against the most common pathogenic agents, such as *S. aureus* is being carried out. Ma et al. [30] performed an investigation on the combined vaccine against *S. aureus* and *S. agalactiae*, infections consisting of FnBPA, ClfA (clumping factor A), antigens representative of *S. aureus* and Sip (Surface immunogenic protein)—and GapC (glyceraldehyde-3-phosphate dehydrogenase C), specific to *S. agalactiae*. Chimeric proteins such as FC (FnBPA + ClfA) and FCGS (FnBP + ClfA + GapC + Sip) have been shown to demonstrate an ability to induce antibody production in mice and lead to a significant reduction in bacterial load in their livers and spleens. FnBP was also the aim of the investigation carried out by Mamo et al., who observed that the introduction of FnBP in mice led to a decrease in the number of *S. aureus* bacteria obtained from the mammary glands and resulted in a significant reduction in cases of severe mastitis [31]. In turn, Zhou et al. showed that a vaccine consisting of a fusion protein named Cna-FnBP (collagen-binding protein-fibronectin binding protein) can be considered a promising candidate for a vaccine protecting against *S. aureus* infection [32]. Promising results were also obtained in another study, in which pregnant heifers were vaccinated with a DNA vaccine containing *fn*BP and *clf*A genes. After challenging the animals with *S. aureus*, the vaccinated heifers demonstrated a decrease in the incidence of mastitis compared to the unvaccinated animals [33].

In immunoblotting performed with monoclonal anti-FnBPA antibodies, the protein of interest whose molecular mass is approx. 120 kDa was detected in the membrane at a size corresponding to ~45 kDa. We hypothesized that it can be related to the structure and molecular mass of the FnBPA protein, which was moving through the polyacrylamide gel during electrophoresis and was subjected to partial protein breakdown; it was tested through a number of experiments, including protein sequencing by MALDI TOF, 2-dimensional electrophoresis, during which we were unable to detect this protein at the height corresponding to ~120 kDa. Therefore, we assumed that a band corresponding to ~45 kDa constituted the part of the protein that included immunoreactive epitopes reactive with antibodies.

To the best of our knowledge, this well-known immunoreactive FnBPA protein has not been described in the context of potential biomarkers in the immunodiagnostics of bovine mastitis caused by *S. aureus*, either in Poland or worldwide. We are aware that our study has some limitations, particularly the number of bacterial strains included in the investigation, which may lead to difficulties in determining the protein conservativity for this protein in the entire population. However, this resulted from the framework of the project under which the research was carried out. FnBPA had been studied in detail by using bioinformatic tools; therefore, a limited number of bacterial isolates was used for the confirmation of in silico analysis. Without a doubt, further investigation with a larger number of samples is essential to confirm the statistical significance of the specificity and sensitivity of FnBPA as a biomarker in immunodiagnostic assays for detecting bovine mastitis caused by *S. aureus*. However, we believe that due to the lack of similar data, our results will expand the knowledge in this area and encourage further investigation on FnBPA in this context.

## 4. Materials and Methods

### 4.1. Materials

The investigation included four representative strains of *S. aureus* isolated from bovine milk with diagnosed mastitis. The strains were obtained from the collection of the West Pomeranian University of Technology in Szczecin, Poland (N =3) and of the Chair of Microbiology, Jagiellonian University Medical College in Krakow, Poland (N = 1). All isolates were stored in a Microbank™ Preservation System (BioMaxima S.A., Lubin, Poland) at −80 °C for further investigation.

Monoclonal antibodies (anti-FnBPA) were produced from BIOLIM S.A. (Gdynia, Poland) in a mouse model. Due to the difficulty obtaining mAb directed against whole FnBPA, the protein monoclonal antibodies used in the experiments were directed against a fragment of this protein (189–507 aa). Therefore, the band of interest was expected at approx. 40 kDa. 

The negative control consisted of milk samples (N = 12) and serum samples (N = 12) obtained from healthy cows (with no clinical signs of mastitis and without bacterial growth in Columbia Blood Agar [Difco, Beirut, Lebanon] and CHROMagar Mastitis [Graso, Krąg, Poland]). Detailed description of samples consisting of negative control had been described in our previous paper [34]. Briefly, samples were collected by veterinarians during routine examinations to determine typical biochemical parameters in healthy animals. Their health status was determined on a basis of physical and biochemical parameters including the number of somatic cells (the average number of somatic cells among the animals was 160,000/mL). The studied herds were assessed by the Polish Federation of Cattle Breeders and Milk Producers, which requires monthly reporting of milk parameters, such as the number of somatic cells per milked animal.

### 4.2. Bioinformatic Analysis

The protein sequences of several bacteria commonly associated with bovine mastitis—including *S. aureus*, the most important bacterium in this study, were subjected to bioinformatic analysis. In order to investigate the highly specific surface proteins representative of *S. aureus* which are potentially found in cow’s milk, and which could be used as specific biomarkers in the immunodiagnostics of bovine mastitis, the following analyses were performed.

**Figure 4:**
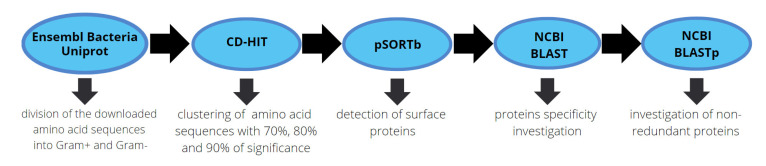
Scheme of the bioinformatic study that led to the identification of FnBPA for *S. aureus*.

In the first step, all amino acid sequences were downloaded from the Esembl Bacteria database [35] and the UniProt database [36]. Next, the amino acid sequences were grouped using the program CD-HIT [37]. The amino acid sequences were then divided into two groups, according to cell surface properties: “positive” and “negative”. CD-HIT analysis was performed for 70%, 80%, and 90% identity thresholds. The surface proteins were predicted using the program PSORTb [38], which allowed us to locate the protein within the bacterial cell. The sequences of the amino acids were classified as “Cellwall”, “CytoplasmicMembrane”, “Extracellular”, and “Unknown”. Only the sequences identified as “CellWall” were chosen for further study. Next, the results from PSORTb and CD-HIT were combined. The surface proteins identified as representative of *S. aureus* were rejected if they were classified into the same sequence cluster as sequences from other Gram-positive species. The remaining proteins were subjected to analysis in BLAST from the National Center for Biotechnology Information (NCBI) [39] and compared with the original database of reference sequences representing several species of bacteria, viruses, and fungi (15 species) that can be found in cow milk (Supplementary Materials). Only those sequences that had no BLAST score and thus were not identified with the reference sequences in any way were selected for further analysis. In the last step of bioinformatic analysis, the amino acid sequences which were not excluded in the previous steps were subjected to analysis in the BLASTp database in order to detect non-redundant protein sequences. As a result of the analysis, only those sequences that were unique for *S. aureus* (with a maximum identity threshold of 75%) were selected (Figure 4).

In order to investigate the structure, function, and immunogenicity of FnBPA, multiple bioinformatic analyses were performed, including the prediction of subcellular localization, signal peptides, antigenicity, and identification of classical or nonclassical. In addition, a selected protein was subjected to linear B cell epitopes and conformational B cell epitope identification, and a PDB model search. The subcellular location of the protein was predicted with PsortB 3.0 [38] and the Bologna Unified Subcellular Component Annotator (BUSCA) server (http://busca.biocomp.unibo.it/ (accessed on 16 December 2022)), separately for *S. aureus* bacteria. The subcellular localizations were labeled with GO annotation.

The first predictions included the identification of signals in the protein sequence and the classification as classical proteins (PRED-LIPO [40], SignalP [41]). Classical protein function was predicted with default scores of 0.45. To identify the antigenicity of FnBPA, in silico prediction was performed using the software program VaxiJen [42]. The antigenic prediction was adjusted with a default score of 0.4, while a protein with a score of ≥0.4 was considered antigenic. The B-cell epitopes of the antigen protein were identified using Antibody Epitope Prediction in the IEDB database [43]. Conformational B cell epitopes were identified through the following steps: a PDB template was identified for the protein sequence using SWISS-MODEL Workspace and including PDB identifiers, then the best template (with the highest identity) was selected to build the model. The selected PDB identifiers were then used to predict linear and discontinuous conformational B-cell epitopes using the software program ellipro [44].

### 4.3. Bacterial Cultivation

In order to revive the 4 *S. aureus,* isolates were unbanked and cultivated on Columbia sheep blood agar (Difco, Lebanon) under aerobic conditions for 24 h at 37 °C and then subjected to further investigation. One colony was then collected and cultured in 9 mL of liquid medium TSB (Tryptic Soy Broth) (Difco, Lebanon) at 37 °C for 24 h under aerobic conditions. Milk samples (100 µL) from healthy animals (negative control) were cultivated on Columbia Blood Agar (Difco) and CHROMagar (CHROMagar™) under aerobic conditions at 37 °C for 24 h. The samples were classified as a negative because of the lack of bacterial growth.

### 4.4. DNA Isolation

The isolation of bacterial DNA was carried out using the commercial Genomic Mini Kit (A&A Biotechnology, Gdańsk, Poland) according to the procedure developed by the manufacturer with addition of 0.4 U/µL of lysostaphin (A&A Biotechnology, Gdańsk, Poland). The concentration and purity of the isolated DNA was examined using the NanoDrop apparatus (Thermo Fisher Scientific, Waltham, MA, USA). The DNA was stored at −20 °C for further investigation.

### 4.5. fnbA Gene Detection

The PCR procedure for the *fnb*A gene encoding FnBPA was carried out according to the procedure by Tristan et al. [45] with slight modifications. Amplification of the detected gene was performed using a pair of a primer set that included forward *fnb*A-F (CATAAATTGGGAGCAGCATCA) and reverse *fnb*A-R (ATCAGCAGCTGAATTCCCATT) (Genomed S. A., Warsaw, Poland) [46] at a final concentration of 0.1 μM. The amplification reaction was carried out using a commercial mixture of 12.5 μL of PCR Mix Plus (A&A Biotechnology, Gdańsk, Poland), 7.5 μL of RNase-free water (A&A Biotechnology), 1.25 μL of each primer, and 2.5 μL of bacterial DNA. PCR was carried out using a T100 Thermal Cycler (BIO-RAD, Hercules, CA, USA) with an initial denaturation step (5 min at 94 °C) followed by 30 cycles of amplification (denaturation for 1 min at 94 °C, annealing for 1 min at 55 °C, and extension for 1 min at 72 °C). The reaction was terminated with a 10 min incubation step at 72 °C. The PCR product (128 bp) was analyzed by 60 min electrophoresis on 1% agarose gel (Prona, Gdańsk, Poland).

### 4.6. Protein Isolation

The isolation of surface proteins with a particular emphasis on FnBPA was carried out according to Banner et al. [47]: bacterial isolates were cultivated in TSB for 18 h at 37 °C under aerobic conditions. After adjustment to a concentration of OD_450_ = 1.3 in phosphate-buffered saline (PBS; Gibco, Thermo Fisher Scientific), the strains were washed twice and harvested by centrifugation (4000× *g* for 3 min). The supernatant was then removed, and the pellets were suspended in 300 µL of PBS containing 0.1 mg/mL of lysostaphin (BioMaxima S.A.) and alternatively 1 mM of the protease inhibitor phenylmethylsulfonyl fluoride (PMSF; BIO-RAD) and incubated for 30 min at 37 °C while being shaken. The protein suspension was then harvested by centrifugation (14,000× *g* for 30 min at 4 °C) and the supernatant was collected. The concentration of surface proteins was estimated using a Pierce™ BCA Protein Assay Kit (Thermo Fisher Scientific). The prepared samples were aliquoted and stored at −20 °C for further analysis.

### 4.7. SDS-PAGE and Immunoblotting

The Laemmli procedure [48] was used 10 µL of the surface protein mixture. After the sample was reduced for 5 min at 100 °C, and centrifuged for 15 min, sodium dodecyl-sulfate polyacrylamide gel electrophoresis (SDS-PAGE) was carried out at a constant current of 25 mA for 50 min. Subsequently, the gels were washed three times in diH2O and stained with Coomassie Brilliant Blue (BIO-RAD) according to the manufacturer’s instruction or directly transferred to a polyvinylidene difluoride membrane (Merck Millipore, Darmstadt, Germany) for immunoblotting. Protein transfer was carried out for 90 min at 135 V.

The membranes were blocked in Pierce Protein Free Blocking Buffer (Thermo Fisher) at 22 °C for 1 h on an orbital shaker and then incubated in anti-FnBPA monoclonal antibodies (BIOLIM) diluted in a 1:100 proportion in phosphate-buffered saline with Tween-20 (PBS-T) (Thermo Fisher) for 1 h at 22 °C on an orbital shaker. The membranes were then washed 3 times for 5 min and placed in goat anti-mouse IgG secondary antibodies conjugated with horseradish peroxidase solution (Thermo Fisher) or in sheep anti-bovine IgG secondary antibodies conjugated with horseradish peroxidase (HRP; Novusbio) in PBS-T in a 1:5000 proportion for 1 h of incubation at 22 °C on an orbital shaker. The membranes were then washed 3 times for 5 min and the bands were visualized using a 1-Step TMB Solution (Thermo Fisher) for 20 min. The reaction was stopped by submersing the membrane in diH2O.

## 5. Patents

The presented results constitute the basis of patent proceedings P.442091.

## Data Availability

The data underlying this article will be shared on reasonable request to the corresponding author.

## Supplementary Materials

The following supporting information can be downloaded at: https://www.mdpi.com/article/10.3390/ijms24076569/s1.
