# Supplementary material for: The Two-Track Investigation of Fibronectin Binding Protein A of Staphylococcus aureus from Bovine Mastitis as a Potential Candidate for Immunodiagnosis: A Pilot Study
Source: Int J Mol Sci. 2023 Mar 31;24(7):6569. doi: 10.3390/ijms24076569 (PMC10094982; doi:10.3390/ijms24076569)
Supplement: Supplementary file 1 [file ijms-24-06569-s001.zip › ijms-2290729-supplementary.pdf]

List of reference sequences representing several species of bacteria, viruses, and fungi that can be found in cow milk

**No.**	**Species**
**1**	*Mycoplasma agalactiae*
**2**	*Mycoplasma alkalescens*
**3**	*Mycoplasma canadense*
**4**	*Mycoplasma bovigenitalium*
**5**	*Acholeplasma brassicae*
**6**	*Acholeplasma oculi*
**7**	*Acholeplasma palmae*
**8**	*Acholeplasma laidlawii*
**9**	*Aspergillus spp.*
**10**	*Aspergillus fumigatus*
**11**	*Bovine herpesvirus 4*
**12**	*Candida albicans*
**13**	*Candida glabrata*
**14**	*Cryptococcus neoformans*
**15**	*Saccharomyces spp.*
